# Fine conductive line printing of high viscosity CuO ink using near field electrospinning (NFES)

**DOI:** 10.1038/s41598-023-45083-6

**Published:** 2023-10-17

**Authors:** Md. Khalilur Rahman, Jin-Sol Lee, Kye-Si Kwon

**Affiliations:** 1 Department of Physics, Comilla University, Cumilla, 3506 Bangladesh; 2 Department of Electronic Materials, Devices and Equipment Engineering, Soonchunhyang University, 22, Soonchunhyang-ro, Asan, Chungnam 31538 South Korea; 3 Department of Mechanical Engineering, Soonchunhyang University, 22, Soonchunhyang-ro, Asan, Chungnam 31538 South Korea

**Keywords:** Chemical engineering, Mechanical engineering

## Abstract

Modern printed electronics applications require patterning of fine conductive lines of sufficient thickness. However, the two requirements for pattern width and thickness are a trade-off. To print fine pattern at a micrometer size, the nozzle diameter must be approximately the size of the pattern width, so only low-viscosity inks are used. As a result, the pattern is likely to be very thin and multiple overlapping printing is required for sufficient conductance. In order to use high viscosity ink for fine patterning, near field electrospinning (NFES) is attracting attention because it can print very thin and thick patterns using large nozzles (high-viscosity ink). Until now, silver paste ink has been used for microconductive patterning using electrospinning. However, Ag nanoparticle (NP) inks are expensive. In this study, we report the use of a relatively inexpensive CuO NP ink for electrospinning-based printing. For implementation, the material preparation, printing and post-processing process are discussed. For post-processing, a continuous wave (CW) green laser with a 532 nm wavelength was used to reduce the CuO to Cu and sinter the nanoparticles. After sintering, the 50 μm width and 1.48 μm thick Cu conductive line exhibited a resistivity of 5.46 μΩ·cm, which is 3.25 times of the bulk resistivity of Cu.

## Introduction

Direct printing method using Ag nanoparticle (NP) metallic ink has been used widely in printed electronics applications because it shows good printability, electrical conductivity and chemical stability^1^. So far, the printed Ag patterns have been used for different applications such as solar cells, touch sensors, integrated electrodes, supercapacitors, etc.^2–4^. However, even though the Ag NP ink has oxidation stability, but it has disadvantage of having higher electromigration effects when electrical current with high density passes through the printed track^5^. As a result, sometimes voids and hillocks are formed and crack nucleation might appear in the printed track. Due to these voids and hillocks formation, sometimes electrical circuit failures can be observed in case of using Ag NP ink^6–8^. Furthermore, Ag NP are expensive and recent industries demand the use of low-cost materials without sacrificing device quality^9^.

As an alternative to Au and Ag NP ink, Cu NP ink has been developed because it is inexpensive and has excellent electrical and thermal conductivity^10,11^. Furthermore, Cu printed patterns are quite stable and have low electromigration effects^7,8^. Despite these merits, the practical use of Cu NP ink has been limited because they are easily oxidized under atmospheric conditions. Therefore, inert environments have been used to store, print, and sinter the Cu NP ink, which increases the processing costs^12,13^. To overcome these disadvantages, recently, copper oxide (CuO) NP ink was developed to form Cu conductive patterns^14^. Copper oxide (CuO) is cheaper than other NP inks such as Au, Ag, even Cu. In addition, it has no oxidation issues and no need for vacuum environment.

Recent printed electronics applications have demanded finer patterns with sufficient thickness. However, there are trade-offs between these two requirements of pattern width and thickness when printing methods are considered for realization. Finer patterns require smaller diameter nozzles. In case of small nozzle diameter, low viscosity ink should be used and the fine patterns are unlikely to have sufficient thickness. To use highly viscous ink, the required nozzle diameter should be large enough. In general, the larger nozzle size produces a larger droplet or large feature size or vice versa. To achieve fine patterns using high viscosity Ag nanoparticle ink, near field electrospinning (NFES) has been used^3,15^.

Researchers are currently exploring various approaches to adapt established electrospinning technology for functional material applications in specific target areas^16^. In conventional electrospinning, which incorporates highly viscous inks involving high molecular polymer, the inclusion of high molecular weight polymers (such as PEO) results in a continuous jet that does not disconnect—bearing a resemblance to aspects of near field electrospinning (NFES). However, the pivotal difference is the integration of functional materials into the process. Furthermore, to achieve straight line printing, a short stand-off distance of a few millimeters and a high printing speed exceeding 100 mm/s are essential factors. On the contrary, electrospray entails the atomization of ink droplets and is commonly used for coating applications rather than direct printing. This entails distinctions in parameters such as voltage, stand-off distance, and ink viscosity when compared to electrospinning^17,18^.

Near field electrospinning (NFES) has gained interest as a sustainable material processing technique due to its simple operation and wide adaptability for fabricating eco-friendly fibers at the nanoscale^19–22^. Both functional parameters and spinning solution characteristics are most important in the practical realization of fine line printing.

Depending on the ink, preparation, printing and post-processing methods are different. To authors’ best knowledge, this work is the first published work using CuO ink for NFES printing. In this work, NFES printing using high viscosity CuO NP ink is investigated in detail. After CuO fine lines are printed, Cu conductive lines are formed using a continuous wave laser of 532 nm wavelength. The green laser sintering is effective because CuO NP ink with reduction agents have a high light absorption range below 700 nm^23,24^. Additionally, we have elaborated on the relevance of intense pulsed light (IPL) sintering for larger area sintering^25^. However, it's worth noting that IPL sintering often employs a substantial spot size, typically in the cm^2^ range, which can inadvertently impact and damage unintended areas. As a result, laser sintering presents itself as a promising alternative for localized direct patterning. Moreover, we acknowledge that various thermal-based methods, such as oven, hot plate, and hot blower sintering, pose challenges in achieving efficient CuO to Cu conversion. Therefore, to the best of the authors' knowledge, for the sintering of CuO ink, only the IPL or laser sintering methods are considered suitable. After sintering, the printed Cu conductive lines are characterized to demonstrate the potential application of low-cost devices in printed electronics.

## Materials and methods

### Substrate preparation

**Figure 1:**
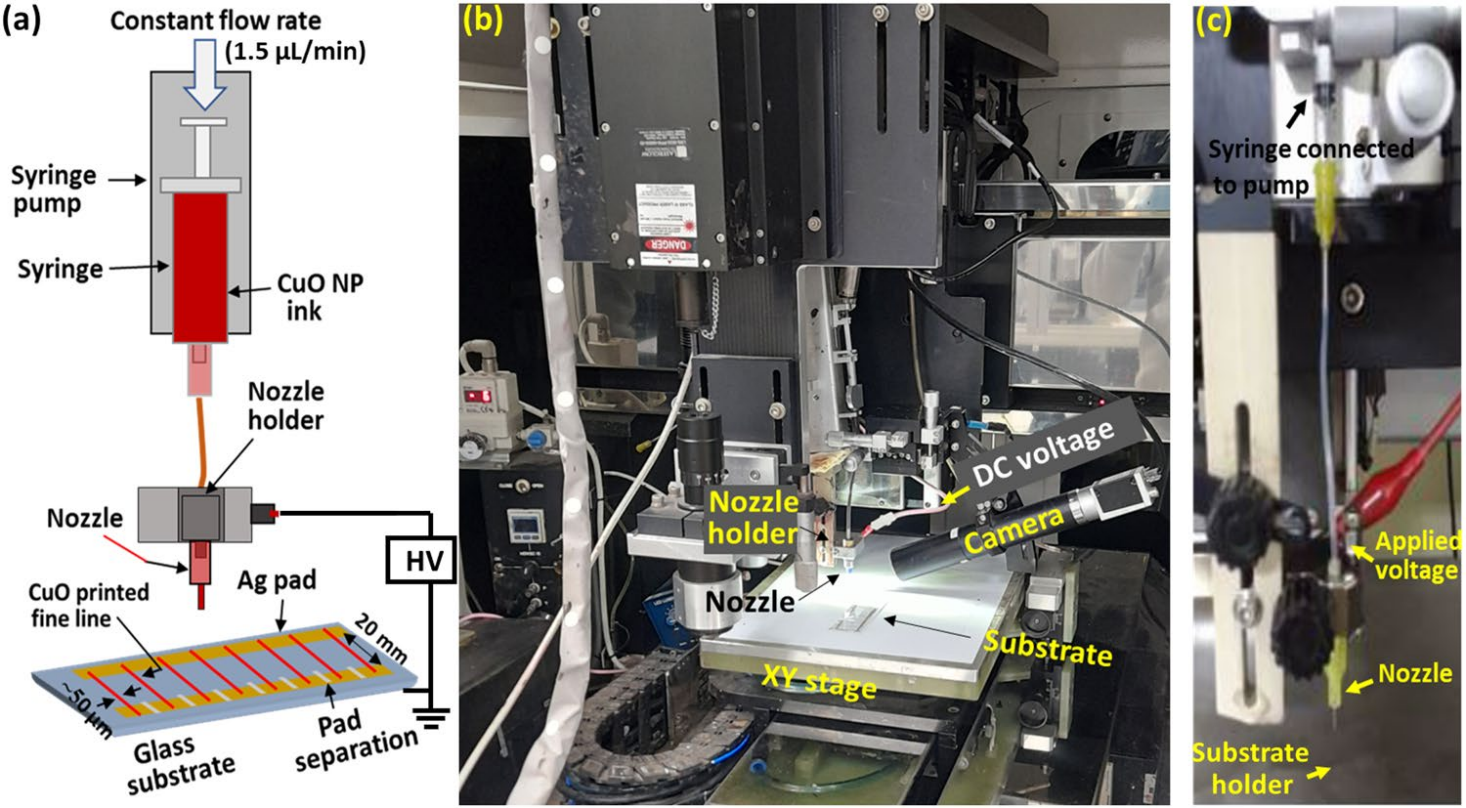
NFES printing system by using CuO NP ink. (**a**) Schematic of the NFES printing for connecting two electrodes on the front side of glass substrate. (**b**) Photo of the NFES printing equipment, (**c**) nozzle component of the equipment.

For experimental demonstration, glass slides of 75 mm in length and 30 mm in width (Microscope slides, Marienfeld Superior, Germany) were purchased. Firstly, the glass substrates were cleaned thoroughly in ethanol using ultrasonic bath and dried with clean compressed air. Two separate conductive Ag pads were fabricated by blade coating method on the front side of the glass substrate. For blade coating, Ag nanoparticle ink (Silverjet DGP 40LT-15C, ANP, South Korea), which contains 30.63 wt% of Ag content, was used. Here, two electrode pads of 50 mm in length and 2 mm in width were fabricated at 20 mm intervals. The electrode pads were sintered on a hot plate at 150 °C for 30 min. After preparing the two conductive electrodes, one of the Ag electrode pads was separated into eight (8) electrodes using an ultra violet (UV) pulsed laser with wavelength of 355 nm (Pulse 355-5, SuZhou Inngu laser, China). Therefore, each single electrode is approximately 6 mm in length and 2 mm in width as shown in (Fig. 1a).

### NFES ink preparation

For the conductive ink for NFES, we used commercially available CuO NP ink (Metalon ICI-021, Novacentrix, USA), which contains 65 wt% CuO with a particle size of around 255 nm. The main solvents of the CuO paste ink are water and ethylene glycol with reduction agents and has a typical viscosity of ~ 207,000 cPs. This CuO NP ink was originally developed for screen printing purposes and suitable for white light photonic sintering, i.e., intense pulsed light (IPL) sintering.

To enhance the stretchability of the conductive ink, necessary for the continuous jetting in the NFES process, CuO NP ink was blended with the polymer solution. The polymer solution was prepared by dissolving 0.3 g of polyethylene oxide (PEO), (M_v_ = 400,000) (MFCD00081839, Sigma-Aldrich, USA) in 10.5 g of mixture solvents composed of ethanol (8.5 g) and deionized (DI) water (2.0 g). To ensure thorough dissolution of PEO with ethanol and DI water, a magnetic stirrer (SP131320-33, Thermo Scientific, China) was employed for mixing over a period of 10 h, leading to the creation of the polymer solution. In the final step, a mixture comprising 10 g of CuO paste ink and 2 g of the polymer solution was blended for 10 min using a vortex mixer (VM-96E, Jeio Tech, South Korea). The whole mixture process was performed at a room temperature of 26 °C with relative humidity of 40%.

The ratio between polymer solution and functional materials involves a trade-off relationship. A smaller amount of polymer solution may impact the jet's stability, while increasing the polymer solution ratio enhances jet stretchability. However, incorporating more polymer could result in a decrease in the content of functional materials and potential conductivity. Thus, optimal ratios need to be determined. The ratio we used aligns with our previous work on Ag ink electrospinning^15^. Our ink preparation guideline aims to minimize polymer solution while maintaining a continuous jet during high-speed printing. To achieve this, we modified the high-viscosity CuO NP ink (~ 207,000 cPs) with 2 g of polymer solutions. While functional materials differ, the polymer solution's role may be analogous to that in Ag paste ink.

In this study, we employed PEO to enhance the stretchability of the ink. A critical aspect was selecting a solvent compatible with PEO melting and CuO inks. Chloroform could be used to dissolve PEO^26^, yet we sought eco-friendly alternatives. Thus, we employed ethanol and DI water to formulate the NFES ink with highly viscous CuO NP ink, considering health and environmental concerns. Note that the main solvents of CuO ink are water and ethylene glycol, which are compatible with ethanol and DI water. This compatibility enhances the suitability of the NFES ink formulation for our approach.

### Printing method of CuO fine lines

The prepared CuO NP ink for NFES was fed to a syringe needle having inner diameter of 370 μm with a constant flow rate of 1.5 μL/min. To supply the ink with constant flow rate, a syringe pump from Nanojet (Nanojet 47963, Chemyx Inc. USA) was used. To produce an electric field for the Taylor cone jet, the nozzle and substrate holders were connected to 1500 V and ground, respectively, which pulls down the charged ink from the nozzle to the substrate, as shown in the schematic of the printing process in Fig. 1a. For this purpose, a high-voltage power supply (SHV30R, ConverTech, South Korea) was used. The laboratory-developed NFES printing system, depicted in Fig. 1b, was employed for printing the CuO fine lines in the experimental setup. Additionally, Fig. 1c illustrates the nozzle assembly of the equipment.

To obtain a uniform printed CuO fine line, we performed idle printing on a paper substrate for more than 30 min, to ensure steady state jetting before printing. While idling printing, printing parameters, such as flow rate (1.5 μL/min), stand-off distance (nozzle tip to substrate distance) (0.6 mm), and high voltage (1.5 kV) applied to the nozzle part, were used to produce the desired steady-state jet stream. After idling printing, the same conditions are utilized for printing CuO fine lines. It's important to emphasize that, even when employing a high voltage of 1.5 kV and maintaining a short distance of 0.6 mm between the nozzle and substrate, no instances of electrical breakdown were encountered. This can be attributed to the substrate's nature; for example, glass with a thickness of 1 mm is not entirely conductive. To obtain straight printed lines along the printing direction, the printing speed was set to 300 mm/s. The printing location was aligned with respect to the electrode pads to connect between the electrodes as shown in Fig. 1a.

### Characterization of sintered Cu conductive line

After printing, the printed lines should be sintered to obtain Cu conductive lines. Prior to sintering, the printed lines were soft baked. Here, two different temperatures of 80 °C and 120 °C were considered to dry out the solvent of CuO NP ink for 1 h. Then, a continuous wave laser with a 532 nm wavelength was used with varying laser powers and scanning speeds.

After sintering, the resistance (R) of the printed lines was measured. The distance between two probes, L, is about 20 mm, and the resistance (R) was measured by multi-meter (TK-4002, Chekman, South Korea). Then the resistivity (ρ) can be calculated using Eq. (1) as

1 $$ \uprho  = {\text{ RA}}/{\text{L}} $$

where, R and A are the measured resistance and cross-sectional area of the sintered line, respectively. The sintered line thickness was measured by 3D surface profiler (ET200, Kosaka Laboratory Ltd. Japan), and cross-sectional microstructure images were taken by using Focused Ion Beam (FIB, Lyra 3, Tescan, Czech Republic). Microscopy (XTCam-D310M, Mitutoyo Measuring Microscope, Japan) was used to investigate the optical microscopic image to understand the sintered line width and overall sintered state.

## Results and discussion

### Selection of nozzle size for NFES printing of CuO ink

**Figure 2:**
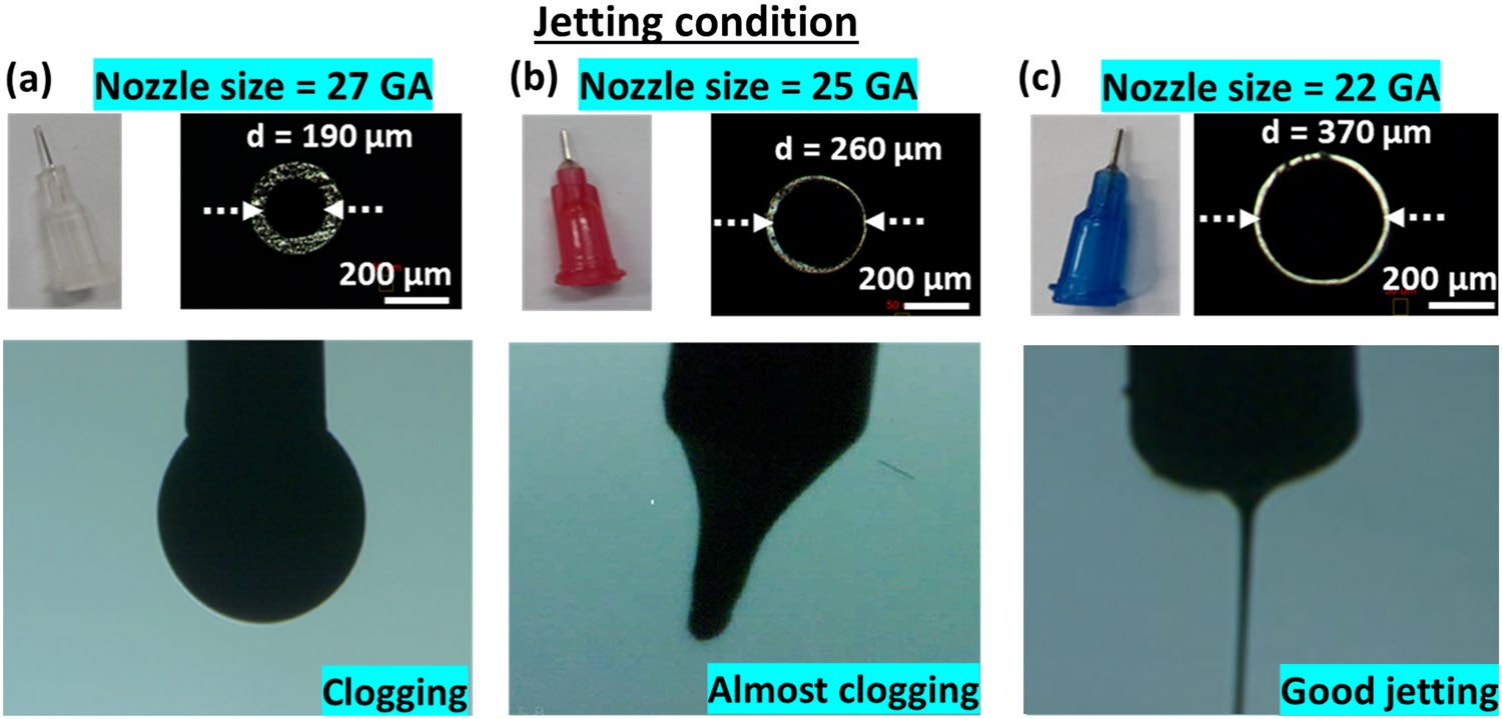
Nozzle inner diameter effect in case of jetting for NFEs printing system. (**a**) No jetting by using nozzle inner diameter of 190 μm, (**b**) near-clogging by using nozzle inner diameter of 260 μm, (**c**) successful jetting by using nozzle inner diameter of 370 μm.

Since the particle size of CuO NP ink is relatively large (~ 255 nm), the selection of the nozzle size (inner diameter) is important. It is always preferred to use smaller nozzle size to achieve fine patterns. However, if smaller nozzles are used, there is concern for nozzle blockage. To investigate the effect of nozzle size, the prepared NFES ink was fed into a syringe needle having different internal diameters of 190 μm, 260 μm and 370 μm at a constant flow rate of 1.5 μL/min as shown in Fig. 2. Figure 2a and b show that the nozzle was clogged when using 190 μm and 260 μm inner diameter nozzles. Considering the nozzle blockage and fine pattern requirement, we used the nozzle with inner diameter of 370 μm and we can get good jetting without any clogging as shown in Fig. 2c.

Figure 2 displays the jet conditions that were measured under static conditions, with no relative motion in relation to the substrate. Its purpose was to illustrate the nozzle size effects, and it was not intended to reflect an actual printing scenario. In the case of using near-field electrospinning, when a charged jet is deposited onto the substrate, it can induce charge repulsion against the incoming jet, thereby contributing to the stability of the jet. Therefore, it's important to note that the jet measured near the nozzle might exhibit slight effects due to the charge repulsion.

To obtain a straight printed fine line using the NFES method, we recommend a printing speed between 100 and 500 mm/s. In this range, the jet tends to deflect towards the moving direction due to the end part being attached to the substrate, and the jet is stretched due to the rapid movement. This phenomenon was discussed in detail in our previous work^16^. In this work, to achieve a straight CuO fine line, we opted for a printing speed of 300 mm/s.

After printing the CuO fine line on the glass substrate, the printed line was pre-baked to reduce solvent from the NFES ink prior to laser sintering. Then, the printed line width was measured by optical microscopy image. In "Surface morphology analysis of CuO fine lines after laser sintering" section, the optical microscopy images provide evidence of successful connectivity between the printed CuO lines and the Ag electrodes on the glass substrate. These images also indicate that the width of the printed lines is approximately 50 μm. The CuO printed line width is uniform throughout the length on glass substrate and also on the Ag pad. To authors’ best knowledge, this is the first results of using electrospinning for fine patterning of high viscosity CuO ink. However, there are trade-off relationship among each parameter, and the ink properties such as particle size should be reduced to further reduce line width. This is beyond the scope of the present work and requires further research.

### Effect of laser power and scanning speed on resistivity of CuO fine lines

**Figure 3:**
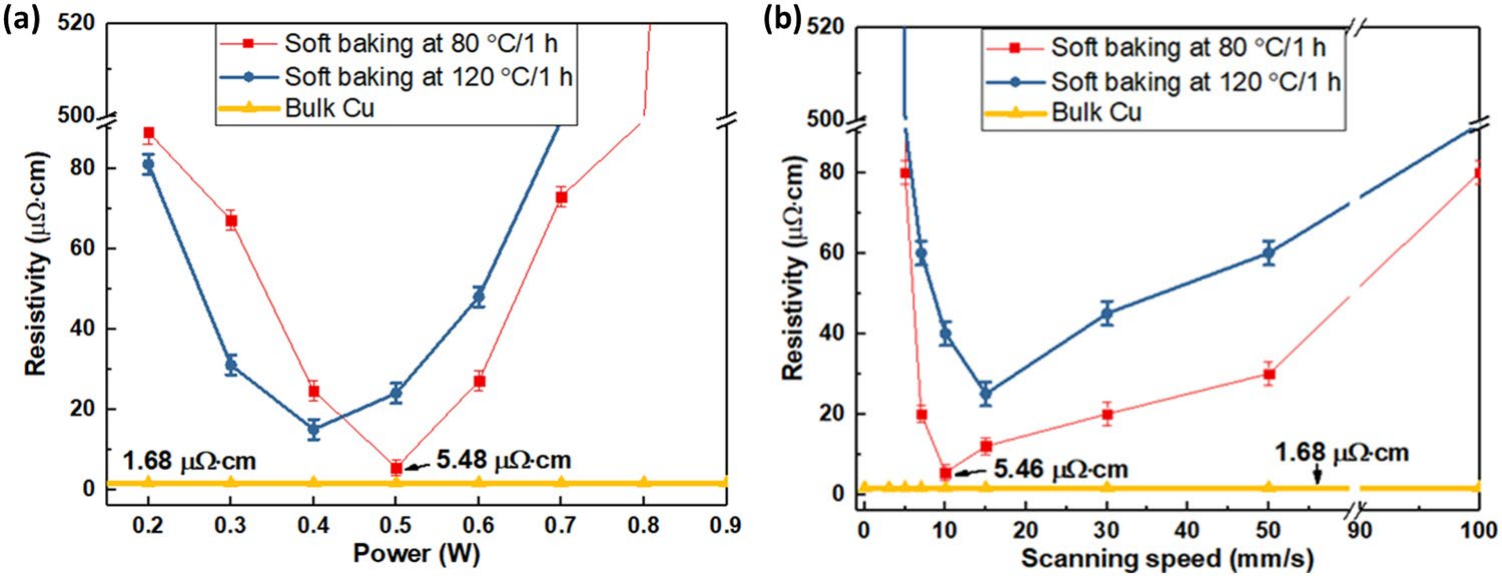
Resistivity measurement of sintered CuO fine line (length 20 mm, width 50 µm). (**a**) Resistivity of sintered CuO fine line as a function of laser power. (**b**) Resistivity of sintered CuO fine line as a function of laser scanning speed.

After subjecting the printed CuO fine lines to pre-baking treatment at temperatures of 80 °C and 120 °C for a duration of 1 h each, the measured resistances were found to be extremely high, surpassing the measurement range limitations of the multi-meter in both instances. This outcome can be attributed to two key factors that render the hotplate heat treatment (at 80 and 120 °C) ineffective in producing conductive lines: (1) The reduction of CuO requires intensive light-based sintering, and (2) the temperature at which nanoparticles sinter is insufficient due to their relatively large size, approximately 255 nm. Consequently, a fusing temperature exceeding 500 °C is necessary for the sintering of such large particles^27^. Consequently, a laser scanning experiment was conducted with a range of laser powers and scanning speeds. As the printed fine line was about 50 μm in width which is smaller than our employed green laser spot size (⁓80 μm) at a laser power of 0.3 W. Therefore, the laser light is irradiated along the printed line of length 20 mm. The laser power and scanning speed are two main important parameters to obtain good conductivity. To investigate the effect of sintering conditions, the resistivity of sintered lines was calculated and plotted as a function of laser power and scanning speed as shown in (Fig. 3a,b). Figure 3a indicates that, to obtain a good conductive Cu fine line, the laser power should be in the range of (0.3 to 0.6) W, while the scanning speed was set to 10 mm/s. The resistivity results show that the laser power of less than 0.3 W was not enough for sintering. On the other hand, when the power exceeds 0.7 W, the irradiated line is damaged.

Therefore, laser scanning experiments were conducted at a power of 0.5 W and at various scanning speeds. The results obtained in Fig. 3b indicate that, to obtain a Cu conductive fine line with low resistivity, the laser scanning speed should be between (5 to 30) mm/s. By adjusting laser sintering conditions, we demonstrate that the resistivity of sintered CuO line could be reduced to as low as 5.46 μΩ cm, which is about 3.25 times that of bulk copper (bulk copper, ρ = 1.68 μΩ cm) as shown in (Fig. 3). It's crucial to mention that the measured printed line exhibits a width of approximately 50 μm and a thickness of 1.48 μm. The recorded resistance for a line length of 20 mm was found to be 17.3 Ω. These dimensions were determined using a 3D surface profilometer for width and a Focused Ion Beam (FIB) investigation for thickness. Further elaboration on these measurements can be found in "Surface morphology analysis of CuO fine lines after laser sintering" and "Thickness of printed CuO fine line" sections. it's important to highlight that prior to assessing the resistivity of the printed line, we consider its shape to be that of a dome or sphere, as depicted in the images. This consideration leads to a calculated cross-sectional area of approximately 6.31 × 10^–11^ m^2^. Subsequently, using Eq. (1), the optimal resistivity of the printed line was computed to be 5.46 μΩ cm. It's important to emphasize that CuO inherently lacks conductivity, and our methodology entails laser sintering to convert CuO into Cu, thus enhancing its conductivity. We acknowledge the significance of assessing conductivity as an indirect measure of process efficacy. The observed reduction in resistivity is influenced by two primary factors. Firstly, the transformation of CuO to Cu significantly contributes to improved conductivity. Secondly, the sintering of Cu particles also contributes to this effect. A lower resistance corresponds to optimal conditions for both of these mechanisms.

The experimental results indicate that the soft baking conditions also play an important role in reducing resistivity. As shown in Fig. 3, the pre-baking temperature of printed CuO fine line was better in case of using 80 °C compared to using 120 °C. The observed superiority of the 80 °C soft baking temperature can be attributed to several factors. Notably, when employing a soft baking temperature exceeding 100 °C, which surpasses the boiling point of water (given that water and ethylene glycol constitute the main solvents of CuO), the solvent within the printed line is prone to evaporating during the soft baking process. Consequently, the residual solvent content within the line diminishes. In this scenario, laser irradiation can induce a significant temperature rise within the line that lacks solvents, thus facilitating faster heat transfer in the thickness direction. This, in turn, can lead to damage in the printed line and a subsequent elevation in resistance. Furthermore, the presence of a small amount of solvent, particularly ethylene glycol, within the printed lines can act as a catalyst in the reduction process of CuO to conductive Cu. This catalytic effect enhances the efficiency of the reduction mechanism.

### Surface morphology analysis of CuO fine lines after laser sintering

**Figure 4:**
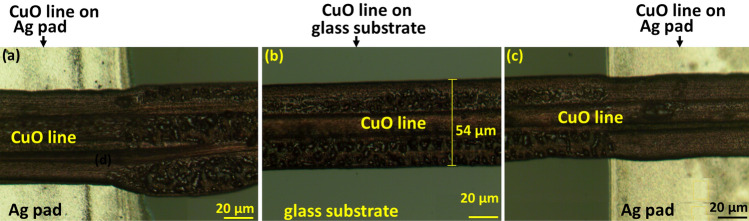
Optical microscopy images of CuO fine line after green laser sintering at power 0.5 W and scanning speed of 8 mm/s. (**a**) CuO line on Ag pad in the left side of glass substrate, (**b**) CuO line on the middle of glass substrate, (**c**) CuO line on Ag pad in the right side of glass substrate.

Figure 4a–c shows optical microscopy images of CuO fine line with green laser sintering at 0.5 W power and 8 mm/s scanning speed. Figure 4a,c depicted a printed line on an Ag pad, while Fig. 4b depicted a printed line on a glass substrate.

**Figure 5:**
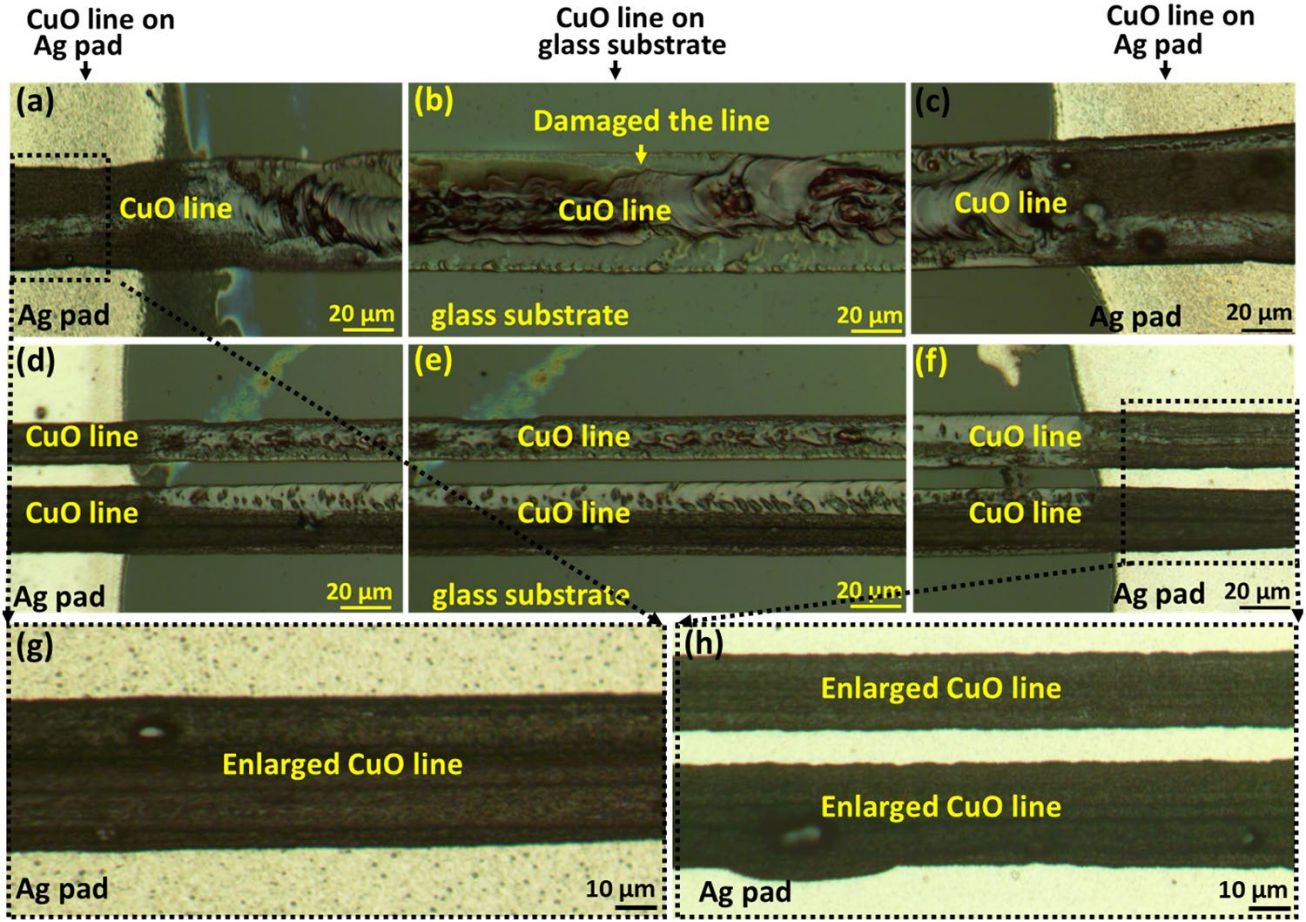
Optical microscopy images of CuO fine line after green laser sintering at power 0.8 W and scanning speed of 8 mm/s. (**a**) CuO fine line sintered on the Ag pad in the left side of glass substrate, (**b**) CuO fine line sintered on the middle of glass substrate, (**c**) CuO fine line sintered on the Ag pad in right side of glass substrate. (**d**–**f**) Two CuO line printed and sintered for connecting between the two electrodes. (**g**) Enlarged CuO fine line on the Ag pad in left side of glass substrate after sintering. (**h**) Enlarged CuO fine line on the Ag pad in right side of glass substrate.

Figure 5 shows the optical microscopy images of the damaged CuO line, when the line was irradiated with high power (0.8 W) of laser light. For better understanding of the sintering status, Fig. 5a–f shows that two different types of lines (Single and double lines) were sintered and both type of lines were damaged. However, it should be noted that the lines on the Ag pad are not damaged as shown in enlarged image of Fig. 5g and h. The possible reason is the thermal conductivity difference of underlying materials. The thermal conductivity of the Ag pad is about (⁓ 418 W/m K), which is significantly higher than that of glass (⁓ 1 W/m K). Similar behaviors is observed in our previous method using electrospinning printing of Ag paste ink on PET film^3^.

### Thickness of printed CuO fine line

**Figure 6:**
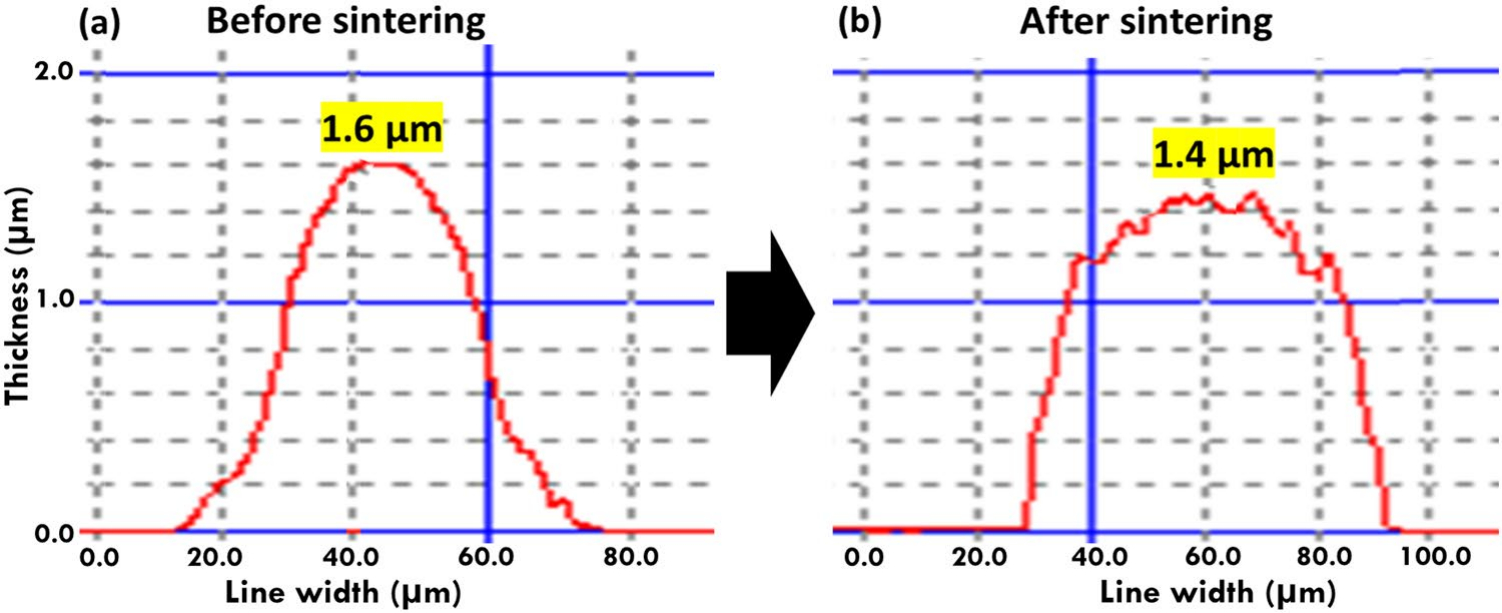
Thickness measurement of the CuO printed fine line by 3D profiler. (**a**) Thickness measurement of CuO fine line before sintering. (**b**) Thickness measurement of CuO fine line after the sintering.

Figure 6 shows the cross-sectional shape of the printed CuO line measured by the 3D surface profiler. As shown in the measured results, the thickness of the sintered printed line is slightly reduced compared to un-sintered line. Figure 6b also indicates that the cross-sectional shape of sintered printed line looks like a dome. The measured thickness is around 1.6 μm for the un-sintered line and 1.4 μm for the sintered line. However, it should be noted that there may be slight errors in case of surface profiler measurement as this is based on measurements using the stylus tip movement along the samples.

**Figure 7:**
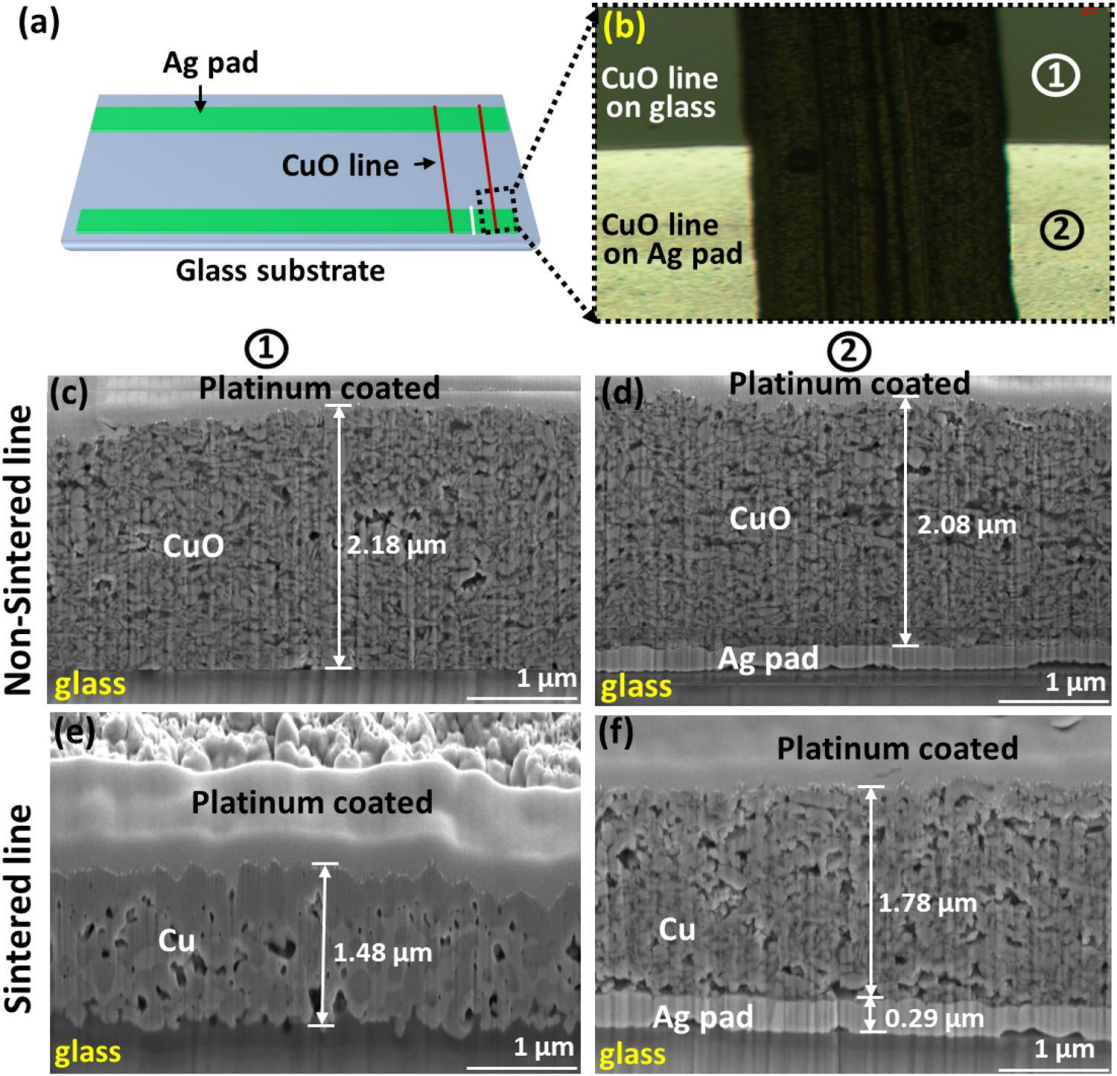
Thickness measurement of CuO printed fine line by FIB investigation before and after sintering. (**a**) Measurement locations of microscopic analysis. (**b**) FIB analysis locations. (**c**) Cross-sectional (FIB) images before sintering at locations (1). (**d**) Cross-sectional (FIB) images after sintering at locations (2). (**e**) Cross-sectional (FIB) images before sintering at locations (1). (**f**) Cross-sectional (FIB) images after sintering at locations (2).

To accurately measure the thickness, the FIB images were investigated as shown in (Fig. 7). The measurement location was indicated in (Fig. 7b). Figure 7c and d show FIB images of CuO line on glass and Ag pad before sintering. As shown in Fig. 7c and d, the CuO particles are not connected one another. On the other hand, when the green laser is irradiated on the surface of the printed line, the reduced Cu particles are properly fused and connected. The connections were different according to the underlying materials. For example, the Cu particles on the Ag pad are slightly less fused as shown in (Fig. 7f). The difference of sintering performance according to location is due to thermal conductivity of underlying materials. Nonetheless, in the case of CuO line thickness reduction, it is evident that exposure to laser light triggers effective fusion among the Cu particles, resulting in the creation of a densely compacted Cu conductive line. This compaction process is intimately linked to the phenomenon of pore annihilation. The observed degree of thickness reduction, approximately 32% (e.g., from 2.18 to 1.48 µm), in comparison to unsintered lines, can be attributed to the compaction of converted Cu particles and the elimination of existing pores.

Therefore, when aiming to control thickness, it is crucial to acknowledge an inherent thickness reduction of approximately 32% as a fundamental aspect of the printing process. However, it is important to note that the extent of thickness reduction is intricately connected to the specific sintering conditions that are applied.

### Simulation of heat distribution behavior after laser irradiation on printed lines

The mechanism of light absorption in CuO is not within the scope of this study due to its complexity and its lack of relevance to our focus. Our aim is to comprehend the heat transfer effects on various underlying materials.

When considering heat transfer, the thermal conductivity of the underlying materials becomes a critical determinant for achieving desirable sintering results with CuO NP ink. This is especially relevant for laser sintering of metal nanoparticles, given that the laser light penetrates only a few dozen nanometers^28^ into the deposited ink layer during the irradiation process. In this scenario, heat transfer plays a crucial role. By incorporating heat transfer considerations, the remaining thickness of the ink experiences sintering through the process of thermal diffusion, extending until it reaches the bottom side of the printed line. Consequently, the sintering outcomes are susceptible to variation based on the thermal conductivity of the substrate (or materials) beneath the printed line.

**Figure 8:**
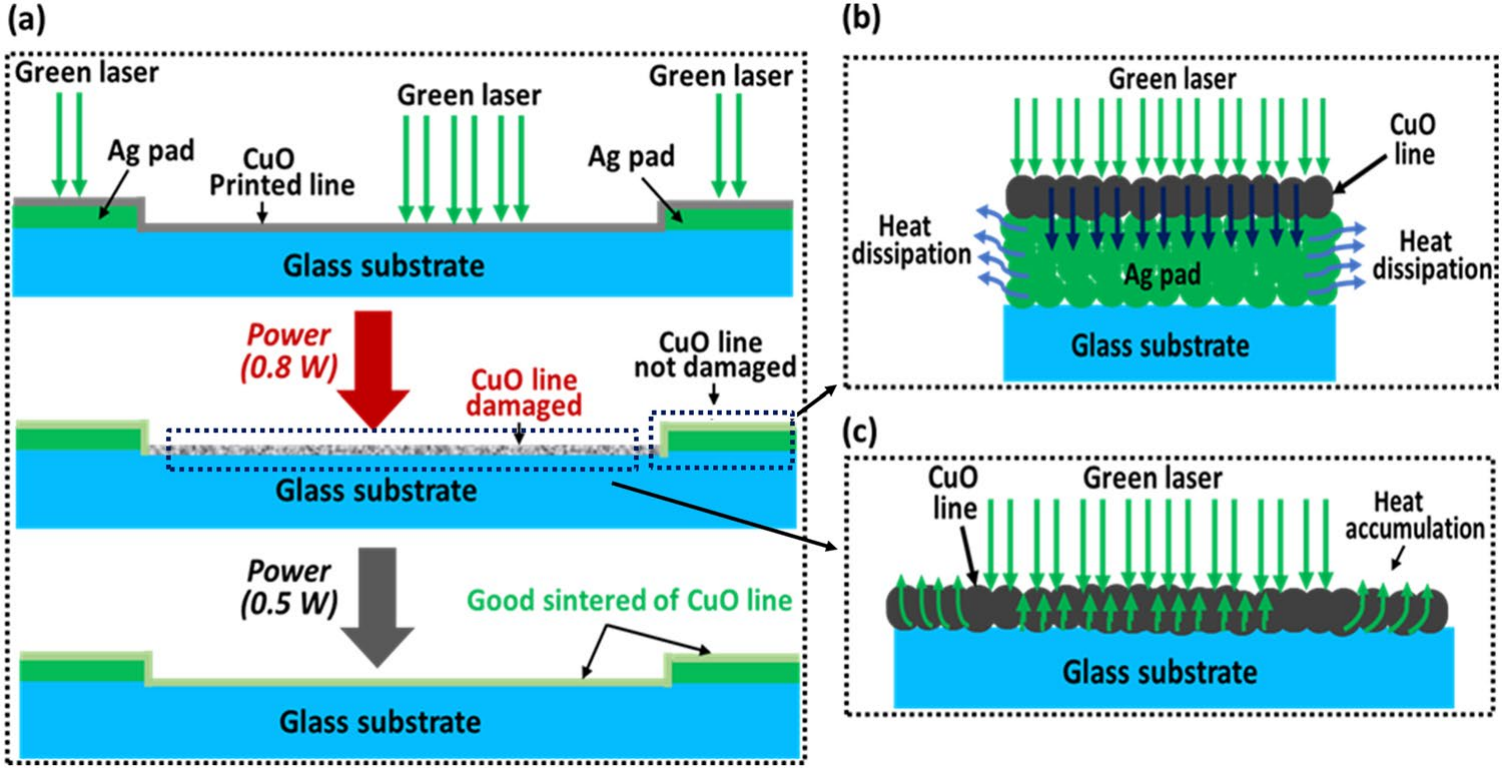
Schematic of the heat distribution process through the CuO fine line, when the line is on Ag pad and also on glass substrate. (**a**) Sintering process with different laser power. (**b**) At high power of laser, the CuO line is not damaged on the Ag pad but (**c**) the CuO line is damaged on the glass substrates due to heat accumulation on the glass substrate.

In Fig. 8 illustrates the heat distribution behavior according to underlying materials, i.e., glass or Ag pad. As illustrated in Fig. 8a, the generated heat on irradiated surface can be transferred through the thickness direction. If the laser irradiates the CuO lines on the Ag pad of glass substrate the additional heat could be easily dissipate through the Ag pad (Fig. 8b) because of its higher thermal conductivity (406 W/m K). On the other hand, when the laser is irradiated on CuO lines printed on the glass substrate, the generated heat is less transferred to the glass (Fig. 8c), because of low thermal conductive glass substrate (1.05 W/m K). which can damage the irradiated printed lines.

**Figure 9:**
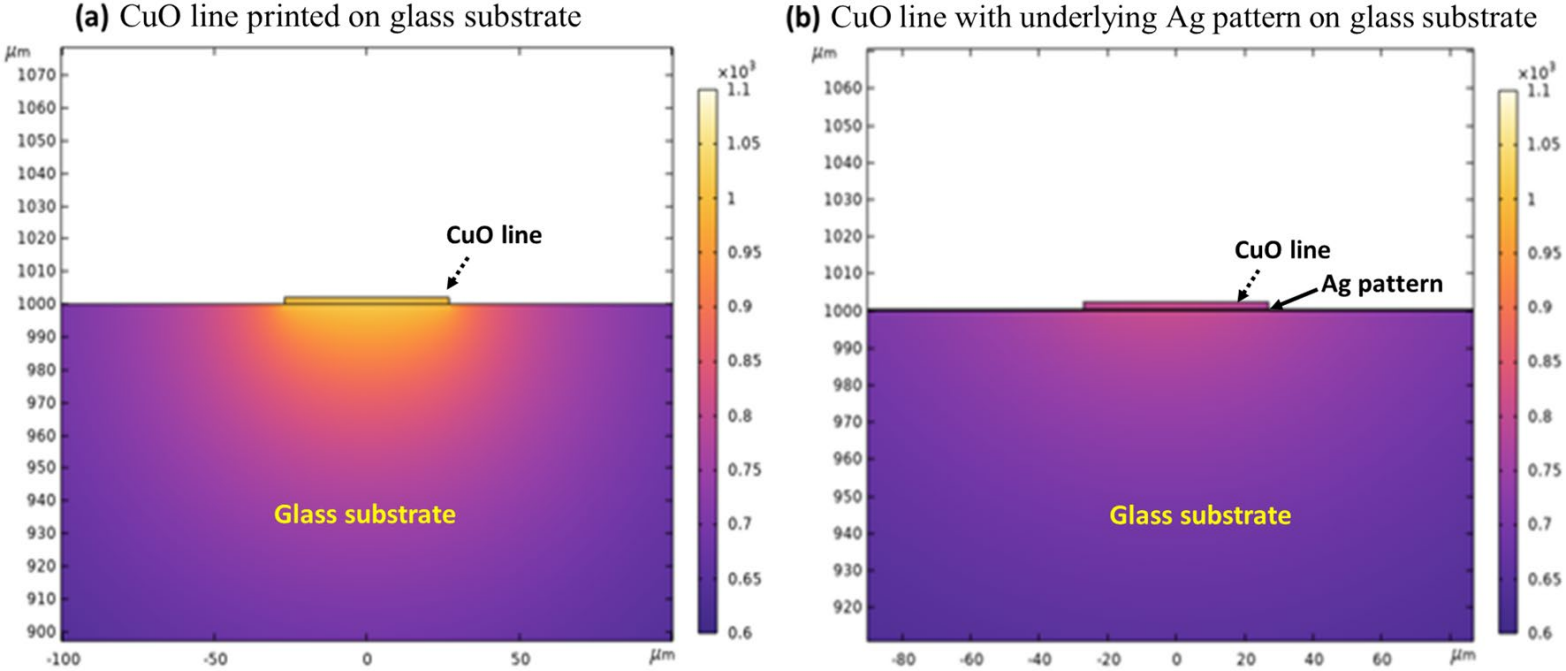
Simulated results of heat distribution after one second of laser irradiation.

For better understanding, we simulated the heat distribution as shown in (Fig. 9) using Finite Element Method (FEM) software (COMSOL). In order to compare the heat distribution behavior according to the underlying materials, we consider two cases. In both cases, the CuO line width and thickness were considered to be 54 and 2 μm, respectively. In case 1 (Fig. 9a) the underlying material is glass whereas in case 2 has underlying material of Ag with thickness of 0.3 μm on glass substrate (Fig. 9b). To simulate the heat behavior, the laser penetration depth and temperature of generated heat due to laser irradiation is referenced from^28^. The irradiating spot temperature can reach 1000 °C with a depth of about 150 nm. After 1 s of time noted that the generated heat is still locally existed near the CuO line on the glass rather than dissipated in surroundings as shown in Fig. 9a. The accumulated heat can possibly lead to the heat damage on the CuO printed lines (Fig. 5b and e). On the other hand, when the irradiated laser light is on the printed lines on the Ag pad, the simulation results indicate that the generated heat can be dissipated more easily (Fig. 9b).

## Conclusions

In this study, we successfully demonstrated the direct printing of copper (Cu) lines with dimensions of approximately 50 μm in width and 1.4 μm in thickness using the near-field electrospinning (NFES) method, followed by green laser sintering. The selection of an appropriate nozzle diameter is crucial, particularly considering the size of the nanoparticles (NPs) involved. In our investigation, it was essential to use a sufficiently large internal diameter (> 350 μm) to ensure proper jetting, given the relatively large size of the CuO particles (approximately 255 nm). It is important to emphasize that the width of the printed pattern is directly correlated with the nozzle size, even though NFES has the capability to produce finer pattern widths than the actual nozzle inner diameter. By employing a nozzle with a diameter of 370 μm, we were able to achieve a pattern width of 50 μm, accompanied by a thickness exceeding 1.4 μm.

After the printing process and before undergoing laser sintering, we found that a soft baking process conducted at a temperature of 80 °C for 60 min yielded the lowest resistivity values. Through a series of scanning experiments, we determined that the optimal conditions for laser sintering are achieved within a laser power range of 0.4–0.6 W and a scanning speed spanning 5–30 mm/s. The choice of optimal power may be influenced by the thermal conductivity of the underlying materials. Importantly, in our study, we utilized two distinct underlying materials characterized by varying thermal conductivities. Consequently, the laser sintering conditions that yield optimal results may not be equally suitable for both underlying materials. It becomes imperative to carefully select conditions that prevent damage to the CuO fine lines, particularly for the low thermal conductivity material (glass).

## Data Availability

The datasets used or analysed during the current study available from the corresponding author on reasonable request.
